# Capsiate Supplementation Reduces Oxidative Cost of Contraction in Exercising Mouse Skeletal Muscle In Vivo

**DOI:** 10.1371/journal.pone.0128016

**Published:** 2015-06-01

**Authors:** Kazuya Yashiro, Anne Tonson, Émilie Pecchi, Christophe Vilmen, Yann Le Fur, Monique Bernard, David Bendahan, Benoît Giannesini

**Affiliations:** Aix-Marseille Université, CNRS, CRMBM UMR 7339, 13385, Marseille, France; Institut d'Investigacions Biomèdiques August Pi i Sunyer, SPAIN

## Abstract

Chronic administration of capsiate is known to accelerate whole-body basal energy metabolism, but the consequences in exercising skeletal muscle remain very poorly documented. In order to clarify this issue, the effect of 2-week daily administration of either vehicle (control) or purified capsiate (at 10- or 100-mg/kg body weight) on skeletal muscle function and energetics were investigated throughout a multidisciplinary approach combining in vivo and in vitro measurements in mice. Mechanical performance and energy metabolism were assessed strictly non-invasively in contracting gastrocnemius muscle using magnetic resonance (MR) imaging and 31-phosphorus MR spectroscopy (^31^P-MRS). Regardless of the dose, capsiate treatments markedly disturbed basal bioenergetics in vivo including intracellular pH alkalosis and decreased phosphocreatine content. Besides, capsiate administration did affect neither mitochondrial uncoupling protein-3 gene expression nor both basal and maximal oxygen consumption in isolated saponin-permeabilized fibers, but decreased by about twofold the *K*_m_ of mitochondrial respiration for ADP. During a standardized in vivo fatiguing protocol (6-min of repeated maximal isometric contractions electrically induced at a frequency of 1.7 Hz), both capsiate treatments reduced oxidative cost of contraction by 30-40%, whereas force-generating capacity and fatigability were not changed. Moreover, the rate of phosphocreatine resynthesis during the post-electrostimulation recovery period remained unaffected by capsiate. Both capsiate treatments further promoted muscle mass gain, and the higher dose also reduced body weight gain and abdominal fat content. These findings demonstrate that, in addition to its anti-obesity effect, capsiate supplementation improves oxidative metabolism in exercising muscle, which strengthen this compound as a natural compound for improving health.

## Introduction

Obesity has become one of the most important public health problems in developed as well as in developing countries. It has been largely acknowledged as an increasing risk factor for insulin resistance, type 2 diabetes, cardiovascular diseases, stroke and also some types of cancers [1]. The fundamental cause of obesity is an imbalance between energy intake and expenditure leading to weight gain and excessive accumulation of adipose tissue. In that context, the use of natural compounds stimulating energy expenditure, in complement of energy intake reduction, represents an interesting approach for the treatment and prevention of obesity.

Capsiate is a nonpungent capsaicin-related compound extracted from the fruit of the CH-19 sweet pepper wherein it is highly concentrated [2]. Chronic administration of capsiate has been reported to promote fat loss in both humans [3] and rodents [4, 5] in the absence of any adverse effect or toxicological changes [6, 7]. This anti-obesity effect would be mediated by an acceleration of basal fatty-acid oxidation in mitochondria [3, 5, 8]. The exact mechanisms activated by capsiate are still unclear, despite the involvement of energy dissipation through uncoupling processes in adipose tissue and skeletal muscle has been evoked [9]. Oral administration of capsiate has been especially shown to activate transient potential vanilloid 1 (TRPV1) receptor, which in turn increases fat tissues lipolysis via sympathetic nervous system and thermogenesis [10–12].

Mitochondrial oxidative phosphorylation represents an important source of energy in both resting and exercising muscle [13]. Then, in addition to its anti-obesity effect, chronic administration of capsiate could be beneficial for working muscle. Nevertheless, very few studies have investigated so far the effects of chronic administration of capsiate on muscle function and energetics. Two-week daily administration of a high dose of capsiate (100 mg/kg body weight) in rat has been shown to significantly reduce (-60%) basal expression of mitochondrial uncoupling protein-3 (UCP3) [4], a mitochondrial inner membrane protein that is highly and preferentially expressed in skeletal muscle and that plays a major role in muscle energy expenditure through the uncoupling of mitochondrial oxygen consumption by the respiratory chain from ATP synthesis [14]. These changes were associated to the reduction of both intracellular acidosis and phosphocreatine (PCr) consumption in exercising gastrocnemius muscle whereas force-generating capacity remained unchanged, thereby suggesting a lower glycolytic flux and a compensatory higher contribution to ATP production [4]. On the other hand, administration of a lower dose of capsiate (10 mg/kg) for the same duration (2-week) in mice has been related to body fat reduction, acceleration of both oxygen consumption (an index of energy expenditure) and fatty-acid oxidation, and improvement of swimming endurance [5, 15], but contrary to what has been reported previously [4] did not affect UCP3 expression [9]. Overall, the functional and metabolic consequences of chronic administration of capsiate remain conflicting and it cannot be excluded that these discrepancies would be linked to a dose-dependent effect.

The aim of this study was to further investigate the potential dose-dependent functional, anatomical and metabolic consequences of chronic capsiate administration, on the basis of in vivo and in vitro experiments in mice. We have especially tested whether capsiate intake affects bioenergetics in exercising gastrocnemius muscle. For this purpose, animals were administrated daily during two weeks with either vehicle (control) or purified capsiate at low (10 mg/kg body wt) or high (100 mg/kg) concentration. Metabolic fluxes were assessed strictly non-invasively with respect to mechanical performance using MR imaging and ^31^P-MRS and oxygen consumption was evaluated in isolated saponin-permeabilized fibers. The corresponding results were analyzed together with quantification of abdominal fat content using whole-body MR imaging and changes in UCP3 gene expression.

## Materials and Methods

### Animal care and feeding

Forty-one C57BL/6 3-month old male mice (Charles River Laboratory, L'Arbresle, France) were used for these experiments. All animal work and care were performed under the supervision of BG (with personal license 13.164 2008/11/25 covering the experiments reported in the manuscript) and conducted in strict accordance with the guidelines of the European Communities Council Directive 86/609/EEC for Care and Use of Laboratory Animals with the approval of the animal experiment committee of Aix-Marseille University. Animals were socially housed as 4–6 per cage in an environmentally controlled facility (12-h light cycle, 22°C) with free access to food and water until the time of the experiment. At the end of experiments, animals were euthanized by cervical dislocation following isoflurane anesthesia and gastrocnemius muscle were removed for in vitro measurements. All reasonable efforts were made to minimize the number and the suffering of animals.

### Experimental design

Capsiate provided by Ajinomoto (Tokyo, Japan) was synthesized as reported previously [16]. Capsiate vehicle, i.e., a solution containing 0.9% NaCl, 3% ethanol and 10% Tween 80 [8] was used as control solution. On a daily basis and during a 2-week period, animals were administrated control or capsiate (at 10- or 100-mg/kg body weight) solution by stomach intubation using a round-ended needle. Two testing sessions were performed. In the first session, which was conducted at the end of the 2-week treatment period, energy metabolism and function were investigated strictly noninvasively in gastrocnemius muscle using ^31^P-MR spectroscopy and MR imaging. For this purpose, 23 animals were randomly assigned to three groups, i.e., control (*n* = 7), capsiate at 10- (*n* = 8) or 100-mg/kg (*n* = 8). The second session was conducted in three additional groups (vehicle, capsiate at 10- or 100-mg/kg; 6 animals per group). Abdominal fat was quantified using in vivo MR imaging before and at the end of the 2-week period. Animals were then euthanized and both gastrocnemius muscles were removed, dissected free of collagen tissue and surrounding fat. The first gastrocnemius muscle was immediately freeze-clamped with liquid nitrogen-chilled metal tongs for measuring UCP3 gene expression and ATP content. The second muscle was placed in an ice-cold isolation solution (Krebs solution containing 2.77 mM of CaK_2_EGTA, 7.23 mM of K_2_EGTA, 6.56 mM of gCl_2_, 5.70 mM of Na_2_ATP, 15 mM of PCr, 20 mM of imidazole, 20 mM of taurine, 0.50 mM of dithiothreitol, 50 mM of K-methansulfonate; pH 7.1 at 22°C) for analyzing mitochondrial function on permeabilized muscle fibers.

### Noninvasive investigation of gastrocnemius muscle function and energetics

#### Animal preparation

Animals were initially anesthetized in an induction chamber using 1.75% isoflurane in 33% O_2_ (0.2 L/min) and 66% N_2_O (0.4 L/min). The left hindlimb was shaved and electromyography cream was applied at the knee and heel regions for optimizing transcutaneous electrostimulation of gastrocnemius muscle. Muscle contractions were elicited using transcutaneous surface electrodes connected to an electrical stimulator (Type 215/T; Hugo Sachs Elektronik-Harvard Apparatus GmbH, March-Hugstetten, Germany). Ophthalmic cream was applied to protect corneas from drying. Each anesthetized mouse was placed supine into a home-built experimental device allowing the strictly noninvasive MR investigation of gastrocnemius muscle energy metabolism and function [17]. During experiment, anesthesia was maintained with a home-built facemask continuously supplied with 1.75% isoflurane in 33% O_2_ (0.2 L/min) and 66% N_2_O (0.4 L/min). The lower hindlimb was positioned inside a 20 mm-diameter Helmholtz-type imaging coil so that the belly of the gastrocnemius muscle was located above an elliptical (8 x 12 mm) ^31^P-MR spectroscopy surface coil and the foot was placed on the pedal of an ergometer including a force transducer. Pedal position was adjusted for modifying the angle between the foot and the lower hindlimb so that the gastrocnemius muscle was passively stretched at rest in order to produce a maximum isometric twitch tension in response to supramaximal square wave pulses (1.5 ms duration; 15–20 mA). Body temperature was controlled and maintained at a physiological level throughout the experiment using a feedback loop including an electrical heating blanket, a temperature control unit (ref. 507137; Harvard Apparatus, Holliston, MA, USA) and a rectal thermocouple probe.

#### Mechanical performance measurements

The analog electrical signal coming out from the force transducer was amplified (AD620 operational amplifier; Analog Devices, Norwood, MA, USA; 70 dB gain; 0–5 kHz bandwidth) and converted to a digital signal (PCI-6220; National Instrument, Austin, TX, USA) that was continuously monitored and recorded on a personal computer using the WinATS 6.5 software (Sysma, Aix-en-Provence, France). Isometric force production (expressed as force-time integral) was calculated every 15 sec by integrating the absolute isometric tension (in N) with respect to time, and was normalized with gastrocnemius muscle volume calculated from hindlimb MR images (see below).

#### MR data acquisition

MR investigations were done in the 4.7-Tesla horizontal magnet of a 47/30 Biospec Avance MR system (Bruker, Karlsruhe, Germany) equipped with a Bruker 120-mm BGA12SL (200 mT/m) gradient insert. Ten noncontiguous axial imaging slices (1-mm thickness, 0.5-mm spaced) were acquired across the resting lower hindlimb using a RARE sequence (8 echoes; 67.9-ms effective echo time; 16.7-ms actual echo time; 2000-ms repetition time; one accumulation; 20 x 15 mm^2^ field of view; 256 x 256 matrix size). ^31^P-MR spectra (8-kHz sweep width; 2048 data points) from the gastrocnemius region were continuously acquired throughout a standardized rest-exercise-recovery protocol consisting of 6 min of rest, 6 min of repeated maximal isometric contractions electrically induced at a frequency of 1.7 Hz, and 15 min of post-electrostimulation recovery. A fully relaxed spectrum (12 scans, 20-s repetition time) was recorded at rest, and was followed by 768 saturated free induction decays (FID; 1.875-s repetition time). The first 64 FIDs were acquired at rest and summed together. The next 192 FIDs were obtained during the electrostimulation protocol and were summed by packets of 32, thereby allowing a 60-s temporal resolution. The remaining 512 FIDs were acquired during the recovery. The first 224 FIDS were summed as 7 packets of 32; the following 192 FIDs were summed by packets of 64 and the last 96 FIDs were summed together.

#### MR data processing

Imaging and spectroscopy MR data were processed using custom-written analysis programs developed using IDL software (Interactive Data Language, Research System, Inc., Boulder, CO, USA) [18, 19]. For each MR image, region of interest was manually outlined so that the corresponding cross sectional area of the gastrocnemius muscle was measured. Muscle volume was calculated as the sum of the six volumes included between seven consecutive slices. Relative concentrations of phosphorylated compounds were obtained from ^31^P-MR spectra with a 60-s time resolution by a time-domain fitting routine using the AMARES-MRUI Fortran code and appropriate prior knowledge for the ATP multiplets [20]. Absolute concentrations were expressed relative to a resting -ATP concentration measured in vitro using high-performance liquid chromatography (see below). Intracellular pH (pH_i_) was calculated from the chemical shift of the P_i_ peak relative to that of PCr [21].

#### Metabolic fluxes calculation

ATP production rates from oxidative and anaerobic pathways were calculated in contracting gastrocnemius muscle according to the quantitative interpretation of bioenergetics data [22–24].

Oxidative ATP production rate (*Q*) was calculated considering that ADP stimulates mitochondrial ATP synthesis through a hyperbolic function [25]: *Q* = *Q*_max_/(1+*K*_*m*_/[ADP]), where *Q*_max_ is the maximal oxidative ATP synthesis capacity and *K*_*m*_ is the ADP concentration at half-maximal rate of oxidative ATP synthesis. *K*_*m*_ was determined for each group from oxygen consumption measurement in permeabilized fibers (see below). ADP concentration was calculated from [PCr], [ATP] and pH_i_ considering the equilibrium constant (*K* = 1.67 10^9^ M^-1^) of the creatine kinase (CK) reaction [26]. *Q*_max_ was calculated using the initial rate of PCr resynthesis at the start of the recovery period (*V*PCr_rec_) and ADP level at the end of the electrostimulation protocol: *Q*_max_ = *V*PCr_rec_ (1+*K*_*m*_/[ADP]_end_). *V*PCr_rec_ was the product of *k*_rec_ (the pseudo-first-order rate-constant of PCr recovery) and ΔPCr (the amount of PCr consumption measured at the end of the electrostimulation protocol). In order to determine *k*_rec_, the time-course of post-electrostimulation PCr resynthesis was fitted to a first-order exponential curve with a least mean-squared algorithm: *k*_rec_ = -[ln(PCr_t_/ΔPCr]/t.

Anaerobic ATP production was calculated as the sum between ATP produced by PCr degradation via CK reaction and by glycolysis. The rate of ATP production from the CK reaction (*D*) was directly calculated using the [PCr] time-course throughout the electrostimulation protocol: *D* = -dPCr/d*t*. Glycolytic ATP production rate (*L*) was inferred at any time point of the electrostimulation protocol considering that *L* is related to glycolytic proton generation (*H*_Gly_) with a stoichiometry of 1.5 moles ATP per mole of proton: *L* = 1.5*H*_Gly_ [27]. *H*_Gly_ was calculated from the changes in pH_i_ after correcting by protons (i) consumed by PCr degradation via CK reaction (*H*_CK_), (ii) passively buffered in the cytosol (*H*_β_), (iii) leaving the cell (*H*_Efflux_) and (iv) produced by oxidative phosphorylation (*H*_Ox_): *H*_Gly_ = *H*_CK_+*H*_β_+*H*_Efflux_-*H*_Ox_. Calculation of *H*_CK_ was done from the stoichiometric coefficient (*φ* = 1/(1+10^(pHi-6.75)^) representing the number of protons generated per mole of PCr degraded [28]: *H*_CK_ = *φ*dPCr/d*t*. Proton buffering was the product of the apparent gastrocnemius buffering capacity (*β*_total_, in Slykes, millimoles acid added per unit change in pH_i_) and pH_i_ changes (ΔpH_i_ = pH_observed_-pH_rest_): *H*_β_ = -*β*_total_ΔpH_i_, in which *β*_total_ takes into account the buffering capacity of P_i_ (*β*_Pi_ = 2.3[P_i_]/((1+10^(pHi-6.75)^)(1+10^(6.75-pHi)^) [28]) and the tissue buffering capacity (*β*_tissue_), which varies linearly between pH 7 (16 Slykes) and pH 6 (37 Slykes) in murine gastrocnemius muscle [29]. During the electrostimulation protocol, *H*_efflux_ was calculated from the linear proportionality constant *λ* (in mM/min/pH unit) relating proton efflux rate and pH_i_: *H*_efflux_ = -*λ*ΔpH_i_. This constant was determined at the start of the recovery period (*λ* = -*H*_efflux_/ΔpH_i_) considering that, despite the intracellular proton load associated with aerobic PCr resynthesis, pH_i_ recovers back to its basal value as a result of a net proton efflux from the cell. Hence, *H*_efflux_ can be calculated considering proton production from CK reaction and mitochondrial ATP synthesis and pH changes: *H*_efflux_ = *H*_CK_ + *H*_Ox_ +*β*_total_dpH_i_/d*t*. Proton production by oxidative ATP synthesis was quantified from the coefficient *m* representing the number of proton produced by mole of ATP generated through oxidative phosphorylation [28]: *H*_Ox_ = *mV*PCr_rec_, with *m* = 0.16/(1+10^(6.1-pH)^).

#### Energy cost of contraction

PCr cost of contraction was calculated by scaling the initial rate of PCr degradation (*V*PCr_stim_) at the onset of the electrostimulation protocol to the amount of force produced during the first 15-s interval of the protocol. *V*PCr_stim_ was calculated as the product of *k*_stim_ (the pseudo-first-order rate-constant of PCr degradation) and [PCr]_cons_ (the amount of PCr consumed at the end of the electrostimulation protocol). The constant *k*_stim_ was determined by fitting the time-course of PCr degradation to a single exponential curve described by the equation: [PCr]_t_ = [PCr]_end_+[PCr]_cons_e^-*k*t^, where [PCr]_end_ is the concentration of PCr measured at the end of the electrostimulation protocol. Oxidative and glycolytic costs of contraction were calculated at the onset and at the end of the electrostimulation protocol as the ratio between raw ATP production and force output during the same period of time

### In vivo quantification of abdominal fat depot

Animals were anesthetized as described above, and axial MR images of the abdominal region were acquired using a proton probe (PRK 200 RES 200, Bruker, Karlsruhe, Germany) and a high-resolution 3-D (turbo spin echo) sequence with the following parameters: 5.530-ms echo time; 77.85-ms effective echo time; 300-ms repetition time; 2 averages; 40 x 40 x 40 mm^3^ field of view, 128 x 128 x 64 matrix size. Absolute quantification of abdominal fat depots was processed using a custom-written analysis program developed on the IDL software.

### UCP3 gene expression

To quantify UCP3 mRNA expression, real-time PCR was performed with a Light Cycler 480 SYBR Green I Master (Roche Applied Science France). Eukaryotic 18S rRNA was used as internal housekeeping gene. The specific primers were: UCP-3 forward ^5’^ TAC CCA ACC TTG GCT AGA CG ^3’^ and reverse ^5’^ GTC CGA GGA GAG AGC TTG C ^3’^; 18S forward ^5’^ ACC GCG GTT CTA TTT TGT TG ^3’^ and reverse ^5’^ AGT CGG CAT CGT TTA TGG TC ^3’^. Briefly, total RNA was extracted from freeze-clamped gastrocnemius muscle (RNeasy Fibrous Tissue Mini kit, Qiagen France) and RNA quantity was determined by reading 1 μL of RNA with a NanoDrop ND-1000 spectrophotometer (Thermo Fisher Scientific, France). RNA (1 μg) was reverse transcribed (QuantiTect Reverse Transcription kit, Qiagen France) and the equivalent of 5 ng of initial RNA was subjected to PCR amplification in a 6 μl final volume with 0.5 μM each of the forward and reverse primers. The standard amplification program included an initial 10-min denaturation at 95°C followed by 45 cycles consisting of 10 s at 95°C, 15 s at 60°C and 15 s at 72°C. Melting-curve analysis were done for confirming the generation of specific PCR products. Each sample was run in triplicate with serial dilutions of a cDNA mixture to generate a standard linear curve that was used to estimate relative mRNA expression of UCP-3 normalized with 18S.

### Intramuscular ATP content

Freeze-clamped gastrocnemius muscles (10 to 20 mg) were homogenized in 1.2 mL of ice-cold 0.6 M perchloric acid. After 15-min incubation at 4°C, the homogenates were centrifuged (15 min, 2000 x *g*, 4°C) and the pellets were dissolved into 1 ml NaOH for protein calculation [30]. After pH neutralization with K_2_CO_3_ and 30-min incubation at 4°C, the supernatants were used for ATP content determination with an ion-pairing reverse-phase high-performance liquid chromatography (HPLC) system (Merck-Hitachi L-6200A pump and L-7400 UV-visible detector, Darmstadt, Germany) [31]. Thymidine monophosphate (T7004; Sigma, Poole, UK) was used as an internal standard.

### Mitochondrial respiration on permeabilized fibers

Respiratory parameters of the total mitochondrial population were studied in permeabilized saponin-skinned fibers according to Kuznetsov et al. [32]. Briefly, thin fiber bundles (100–200 μm in diameter) were excised from muscles in isolation solution, and the fibers were manually separated from each other. Sarcolemma permeabilization was done by incubating dissected fibers for 30 min with gentle shaking in the same solution containing 50 mM of saponin. Fibers were then transferred for 10 min into a respiration solution (pH 7.1 at 22°C) containing 2.77 mM of CaK_2_EGTA, 7.23 mM of K_2_EGTA, 1.38 mM of MgCl_2_, 3 mM of K_2_HPO_4_, 20 mM of imidazole, 20 mM of taurine, 0.50 mM of dithiothreitol, 90 mM of K-methansulfonate, 10 mM of Na-methansulfonate, 5 mM of glutamate, 2 mM of malate, 2 mg/ml of fatty acid—free bovine serum albumin. This step was repeated twice in order to wash out saponin and metabolites that could interfere with measurements. Mitochondrial respiration rate was measured at 22°C with a Clark electrode integrated in a water-jacketed oxygraphic cell (Hansatech Instruments Ltd, Norfolk, England) containing 1–2 mg of fibers in 1.5 mL of respiration solution with continuous stirring. ADP sensitivity was measured by stepwise addition of ADP (from 5 to 2000 μM). The apparent *K*_*m*_ for [ADP] was calculated by plotting the ADP-stimulated respiration rate (*V*_ADP_) to a non-linear fitting of the Michaelis-Menten equation. The maximal oxidative capacity (*V*_max_) was calculated as the sum between *r*_0_ (the basal respiration rate measured without ADP addition) and *r*_max_ (the maximal respiration rate reached with cumulative addition of ADP).

### Statistics

All values are presented as means ± SEM. Statistical analyses were performed using JMP software version 9 (SAS Institute Inc., Cary, NC, USA). Two-way (group × time) analysis of variance (ANOVAs) with repeated measures on time were used to compare the changes during the 6-min electrostimulation protocol for isometric force production, metabolites levels and pH_i_. Other differences were tested using one-way (group) ANOVAs. When a main effect or a significant interaction was found, Tukey post-hoc multiple comparisons test was used for determining pairwise time-points differences between groups. For all tests, the level of significance was set at *P* < 0.05.

## Results

### Body weight, food consumption and abdominal fat volume

The effects of 2-week daily administration of vehicle or capsiate on food consumption, body weight and abdominal fat volume are summarized in Table 1. Over the course of the 2-week period, food intake did not differ between groups, but body weight gain and abdominal fat content were both reduced in the group receiving the higher dose of capsiate when compared with control animals.

**Table 1:** Food intake, body weight and abdominal fat gain over 2-week daily administration of vehicle or capsiate.

	Control	Capsiate 10 mg/kg	Capsiate 100 mg/kg
Body weight before treatment (g)	25.3 ± 0.4	25.4 ± 0.4	25.6 ± 0.5
Body weight gain (g)	0.92 ± 0.65	0.32 ± 0.36	-0.41 ± 0.16*
Food intake (g/animal)	39.9 ± 1.5	41.5 ± 2.3	41.2 ± 2.1
Abdominal fat gain (cm^3^)	1.06 ± 0.38	-0.39 ± 0.46	-0.68 ± 0.51*

Values are means ± SEM. *n* = 6 per group.

*Significantly different from control in the same row.

### Muscle volume and mechanical performance

**Fig 1:**
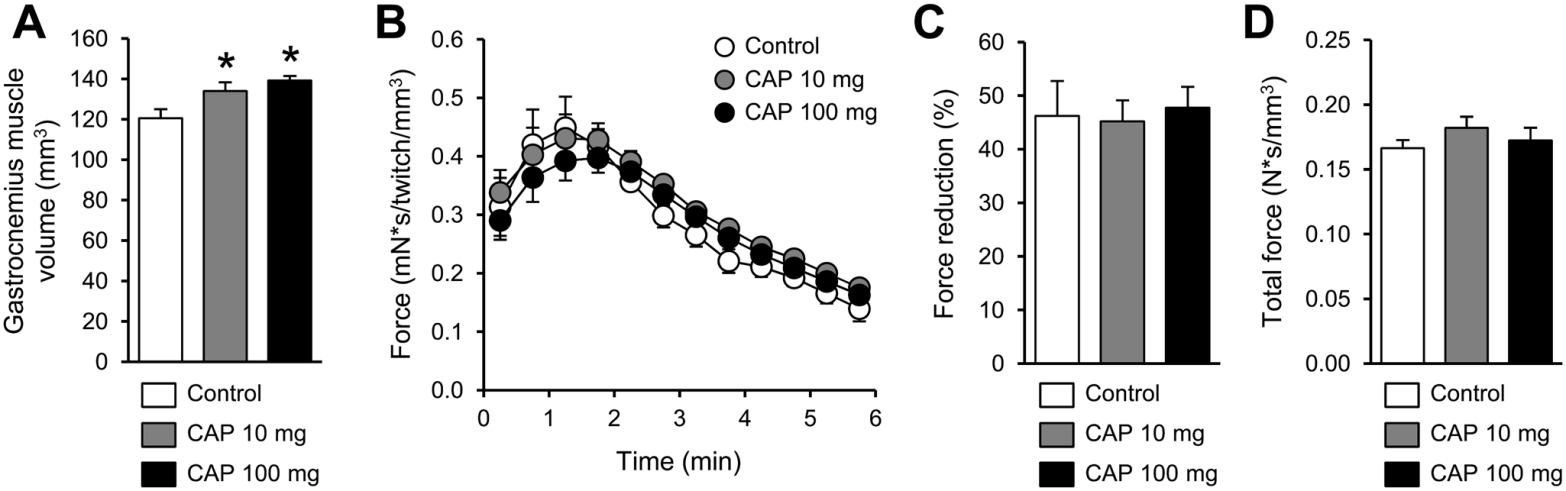
Muscle anatomy and mechanical performance in vivo. Gastrocnemius muscle volume (A) and force produced throughout the 6-min fatiguing electrostimulation protocol performed at the end of 2-week treatment with vehicle (control) or capsiate (CAP) at 10- or 100-mg/kg body weight (B). Extent of force reduction measured at the end of the protocol and expressed as percent of onset value (C). Total amount of force produced during the whole 6-min electrostimulation (D). Data are means ± SEM. * Significantly different (one-way ANOVA; *P* < 0.05) from control.

Gastrocnemius muscle volume (121 ± 5 mm^3^, 134 ± 4 mm^3^ and 139 ± 2 mm^3^ in control, 10-mg and 100-mg capsiate group, respectively) measured from MR images after the 2-week treatment was larger (+11% and +16% in 10-mg and 100-mg capsiate group, respectively) in animals receiving capsiate when compared to control group (Fig 1A). The overall time-course of muscle force during the 6-min electrostimulation protocol (Fig 1B), the extent of force reduction (expressed as percent of onset electrostimulation-protocol value) measured at the end of the protocol (Fig 1C) and the total force developed during the whole protocol (Fig 1D) were not affected by any of both capsiate treatments.

### Metabolic changes

**Fig 2:**
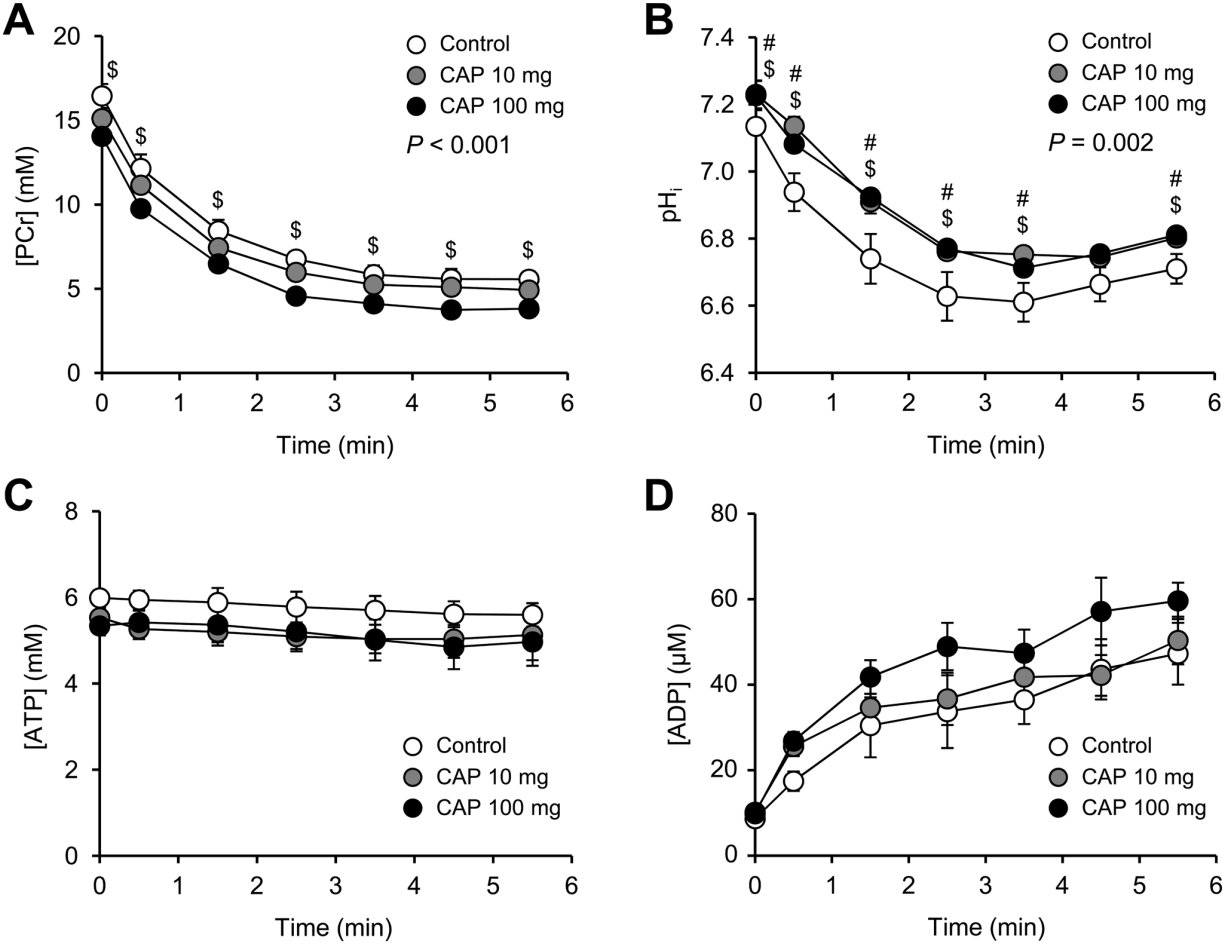
Phosphorylated compounds and pH in mouse gastrocnemius muscle in vivo. Changes in gastrocnemius muscle [PCr] (A), intracellular pH (B), [ATP] (C) and [ADP] (D) throughout the 6-min fatiguing electrostimulation protocol performed at the end of 2-week treatment with vehicle (control) or capsiate (CAP) at 10- or 100-mg/kg body weight. The first time point (t = 0) indicates the resting value. Data are means ± SEM. *P*-value indicates the overall result of the two-way repeated measures ANOVA. When justified (*P*-value < 0.05), Tukey post-hoc multiple comparison was used for determining pairwise differences between groups: ^#^ Significant difference between CAP 10-mg and control. ^$^ Significant difference between CAP 100-mg and control.

In resting muscle, there were no differences among the groups for [PCr]/[ATP] ratio, [ATP] and [ADP] (Table 2). On the contrary, [PCr] was lower (-15%) in the group receiving the higher dose of capsiate compared to control animals, and basal pH_i_ was larger in both groups receiving capsiate (Table 2). At the onset of the electrostimulation protocol, PCr was rapidly degraded (Fig 2A) at a similar rate among the groups (Table 2). After 2–3 min of electrostimulation, [PCr] reached a steady state that remained stable until the end of the protocol (Fig 2A). At that time, the extent of PCr degradation did not differ among the groups (Table 2). It is noteworthy that the initial difference regarding the resting PCr level between control and 100-mg capsiate groups was kept approximately constant during the whole electrostimulation protocol (Fig 2A). The time-course of pH_i_ during repeated muscle contractions was strongly similar among the groups, with a rapid acidosis in the early phase of the protocol, followed by a phase of fairly steady state (Fig 2B). Interestingly, muscle acidosis during the protocol was significantly lower in both groups receiving capsiate (Fig 2B). For each group, ATP level remained close to the basal value during the whole electrostimulation protocol (Fig 2C), whereas ADP level continuously increased (Fig 2D) to reach an end-exercise value that did not differ between groups (Table 2). During the recovery period, PCr, ATP, ADP and pH_i_ progressively reached towards their respective basal value. Importantly, the initial rate of PCr resynthesis was not altered in both groups receiving capsiate (Table 2).

**Table 2:** Effect of 2-week daily administration of vehicle or capsiate on mouse gastrocnemius bioenergetics assessed in vivo using ^31^P-MR spectroscopy.

	Control	Capsiate 10 mg/kg	Capsiate 100 mg/kg
	*n* = 7	*n* = 8	*n* = 8
**Resting period**			
[PCr]/[ATP] ratio	2.75 ± 0.12	2.74 ± 0.07	2.63 ± 0.11
[PCr] (mM)	16.4 ± 0.7	15.2 ± 0.4	14.1 ± 0.6*
[ATP] (mM)	5.9 ± 0.3	5.5 ± 0.2	5.3 ± 0.2
[ADP] (μM)	9 ± 1	11 ± 1	10 ± 1
pH_i_	7.13 ± 0.02	7.26 ± 0.03*	7.23 ± 0.02*
**Onset of the electrostimulation protocol**			
Initial rate of PCr degradation (mM/min)	11.5 ± 1.5	9.6 ± 1.0	10.0 ± 1.0
[ATP] (mM)	5.9 ± 0.4	5.3 ± 0.2	5.3 ± 0.3
[ADP] (μM)	20 ± 4	26 ± 2	27 ± 1
**End of the electrostimulation protocol**			
ΔPCr (relative to basal; %)	66 ± 3	69 ± 2	72 ± 2
[ATP] (mM)	5.9 ± 0.5	5.2 ± 0.6	5.1 ± 0.5
[ADP] (μM)	47 ± 7	57 ± 9	60 ± 4
ΔpH_i_ (relative to basal; pH unit)	0.42 ± 0.04	0.44 ± 0.03	0.42 ± 0.03
**Post-electrostimulation recovery period**			
Initial rate of PCr resynthesis (mM/min)	4.9 ± 0.4	4.4 ± 0.6	3.5 ± 0.4

Values are means ± SEM.

*Significantly different from control in the same row.

### Metabolic fluxes and energy cost of contraction

**Fig 3:**
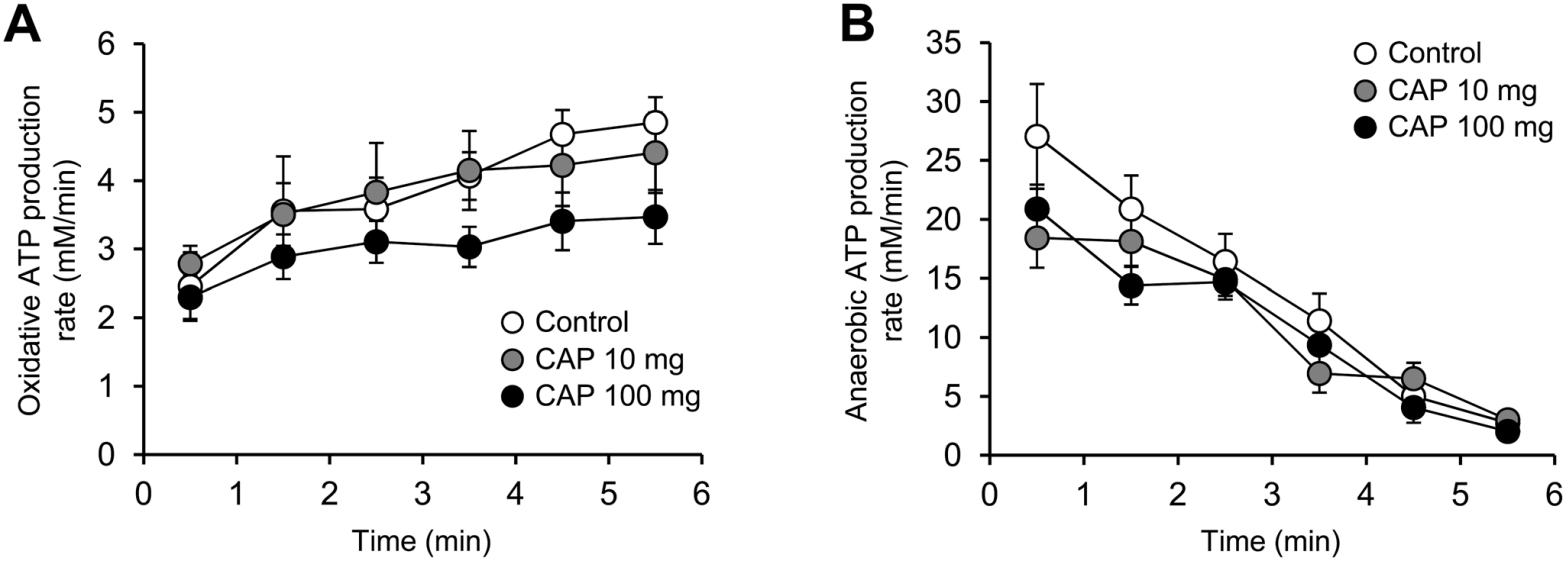
Oxidative and anaerobic ATP productions in contracting mouse gastrocnemius muscle in vivo. Effect of 2-week treatment with vehicle (control) or capsiate (CAP) at two different concentrations (10- or 100-mg/kg body weight) on the time-course of oxidative (A) and anaerobic (B) ATP production rates calculated from in vivo ^31^P-MR spectroscopy data throughout the 6-min fatiguing electrostimulation protocol. Data are means ± SEM. * Significantly different (one-way ANOVA; *P* < 0.05) from control.

**Fig 4:**
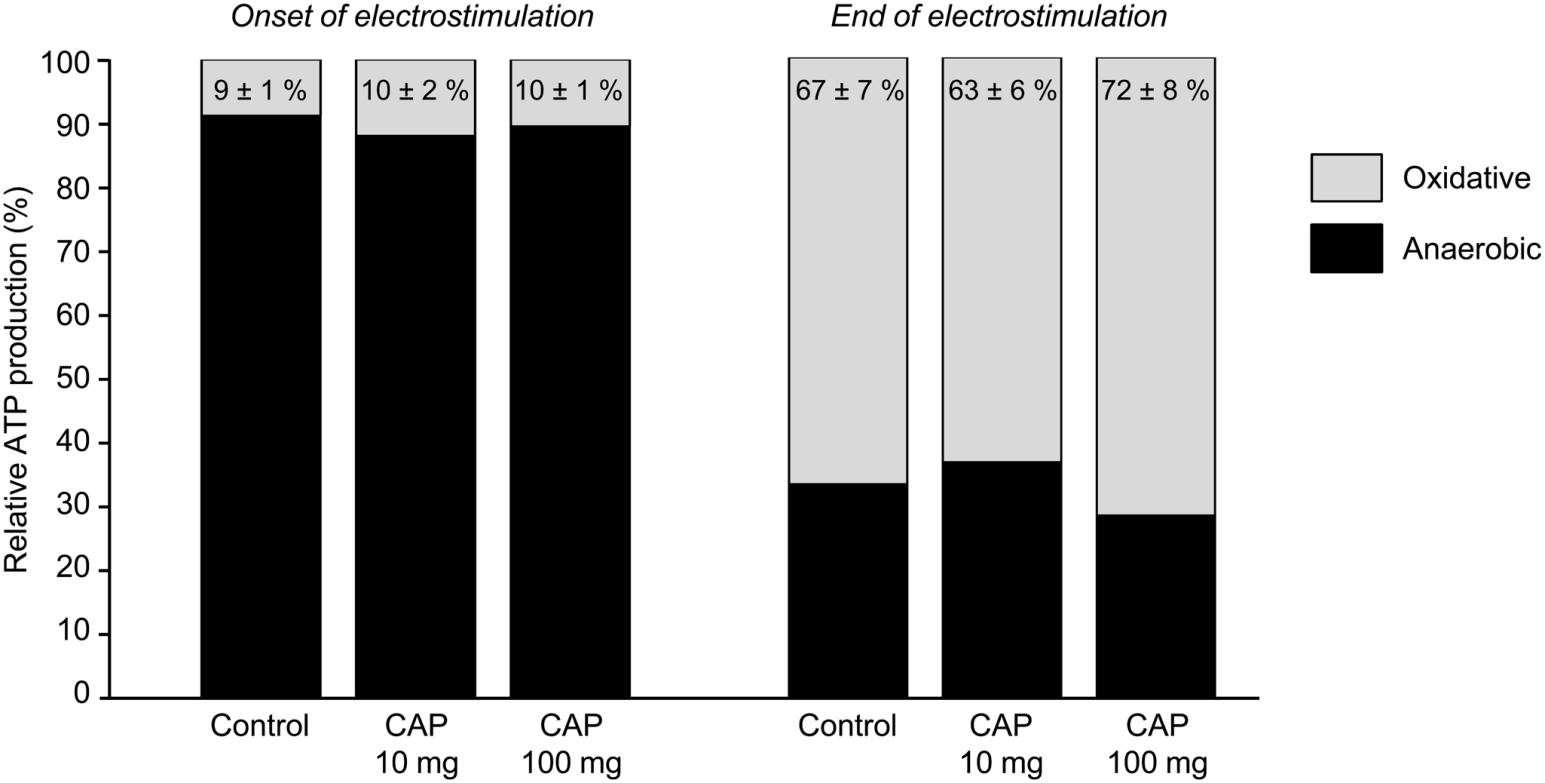
Metabolic fluxes contributions in contracting muscle. Relative contributions of oxidative and anaerobic pathways to total ATP production at the onset and at the end of the 6-min fatiguing electrostimulation protocol performed after 2-week treatment with vehicle (control) or capsiate (CAP) at two different concentrations (10- or 100-mg/kg body weight). Data are means ± SEM.

**Fig 5:**
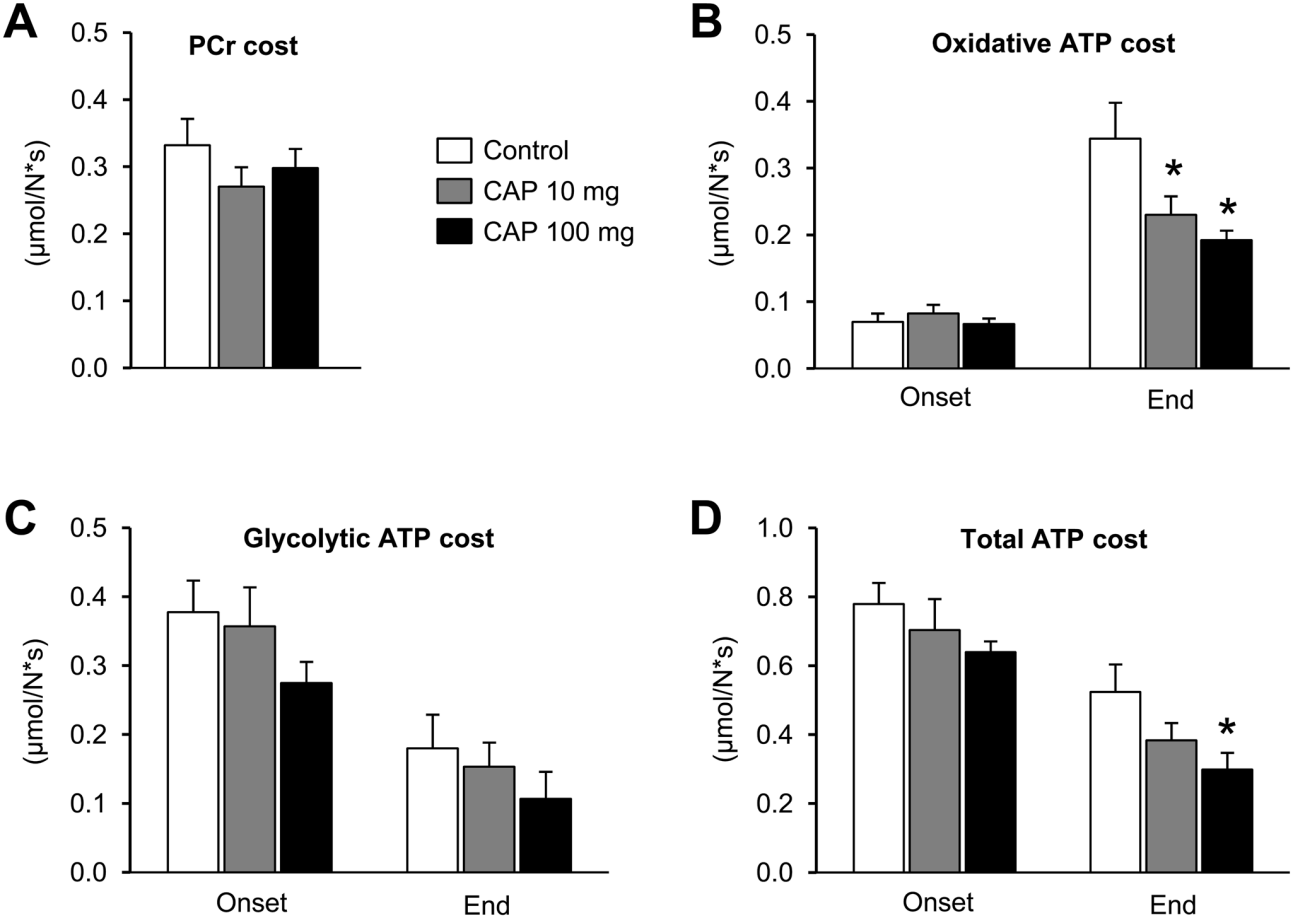
Energy cost of muscle contraction. Effect of 2-week treatment with vehicle (control) or capsiate (CAP) at two different concentrations (10- or 100-mg/kg body weight) on PCr cost (A) and oxidative (B), glycolytic (C) and total (D) ATP cost of contraction at the onset and at the end of the 6-min fatiguing electrostimulation protocol. Data are means ± SEM. * Significantly different from control.

Capsiate treatment did not change the overall time-course of oxidative and anaerobic ATP production rates throughout the electrostimulation period (Fig 3A and 3B). However, oxidative ATP production rate was increased for each group between the onset and the end of the electrostimulation protocol, but this increase was lower in both groups receiving capsiate (-44% and -48%, in 10- and 100-mg groups, respectively) (Fig 3A). Besides, the relative contribution of oxidative ATP production to total ATP production did not differ between groups, averaging ~10% and ~67% at the onset and at the end of the electrostimulation protocol, respectively (Fig 4). There were no differences among the groups for PCr cost (Fig 5A) and oxidative (Fig 5B), glycolytic (Fig 5C) and total (Fig 5D) ATP cost of contraction at the onset of the electrostimulation protocol. At the end of the protocol, oxidative cost of contraction was significantly lower in both groups receiving capsiate (-33% and -44% in the 10-mg and 100-mg group, respectively) (Fig 5B), and total ATP cost was significantly lower (-25%) in the 100-mg group when compared to control (Fig 5D).

### Mitochondrial respiration on permeabilized fibers

Both capsiate treatments did alter neither the basal respiration rate nor the maximal oxidative capacity, but reduced the *K*_m_ of mitochondrial respiration for ADP in the 10-mg (- 56%) and the 100-mg (-64%) capsiate-treated animals when compared to the control group (Table 3).

**Table 3:** Effect of 2-week daily administration of vehicle or capsiate on respiration parameters in isolated permeabilized fibers from gastrocnemius muscle.

	Control	Capsiate 10 mg/kg	Capsiate 100 mg/kg
Basal rate of O_2_ consumption (μmol/min/g)	1.52 ± 0.09	1.82 ± 0.14	1.46 ± 0.16
Maximal rate of O_2_ consumption (μmol/min/g)	3.03 ± 0.29	3.17 ± 0.49	3.01 ± 0.20
*K*_m_ for ADP (μmol/L)	159 ± 29	69 ± 20*	56 ± 10*

Values are means ± SEM. *n* = 6 per group.

*Significantly different from control in the same row.

### UCP3 gene expression

**Fig 6:**
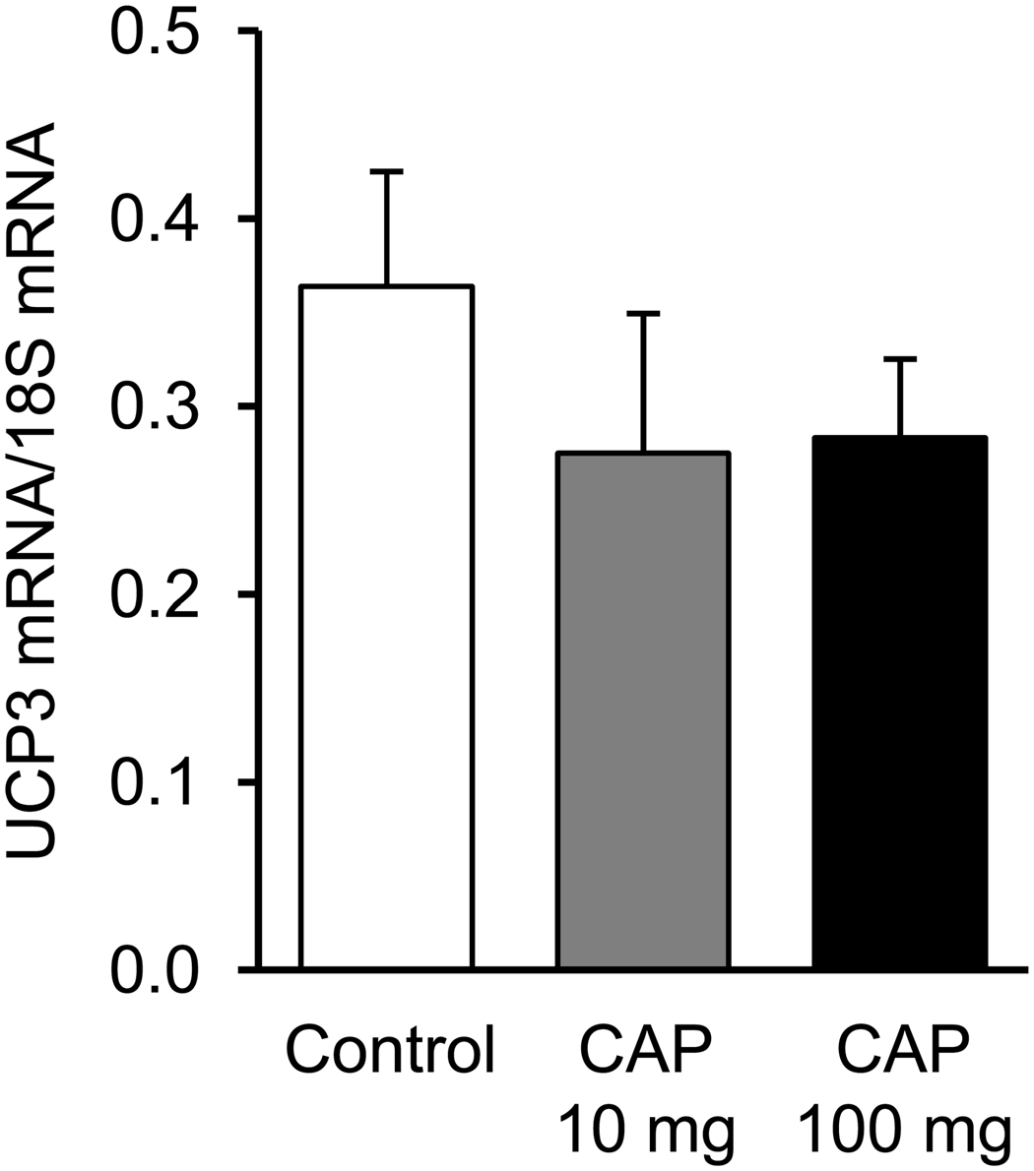
UCP3 gene expression. Basal UCP3 mRNA levels in gastrocnemius muscle normalized to 18S mRNA used as an internal standard in animals daily administered during two weeks with vehicle (control) or capsiate (CAP) at two different concentrations (10- or 100-mg/kg body weight). Data are means ± SEM.

UCP3 mRNA levels in gastrocnemius muscle measured after the 2-week treatment period did not differ among the groups (Fig 6).

## Discussion

The aim of this study was to examine the functional, anatomical and metabolic consequences of chronic administration of a low (10 mg/kg body wt) and a high (100 mg/kg) dose of purified capsiate on mice gastrocnemius muscle. The major findings are that, regardless of the dose, capsiate treatment: (i) did affect neither UCP3 gene expression nor both basal and maximal oxygen consumption in permeabilized muscle fibers, but decreased by about twofold the *K*_m_ of mitochondrial respiration for ADP; (ii) promoted gastrocnemius muscle mass gain; (iii) markedly disturbed basal bioenergetics in vivo; (iv) reduced oxidative cost of contraction; (v) did not affect force-generating capacity nor fatigability. In addition, treatment with the higher dose also reduced body weight gain and abdominal fat content.

Our data show that 2-week daily administration of the higher dose of capsiate reduced body weight gain and abdominal fat content as measured from MR images, which is consistent with previous experiments in human [3] and rodent models [4, 5, 9]. Moreover, we found that these changes were not associated to any reduction of food intake, hence indicating that mice treated with capsiate did ingest the same amount of calories as control animals. Our findings add then further evidence that capsiate suppresses body fat accumulation likely as a result of an increased calories burning.

On the basis of respiratory gas analysis reporting that capsiate supplementation increases resting oxygen consumption, it has been proposed that the anti-obesity effect of capsiate was due to an acceleration of basal fat oxidation [3, 8]. Nevertheless, these measurements were done at the whole-body level and did not provide information related to the specific tissue activated by capsiate although adipose tissue and skeletal muscle have been considered as the most likely candidates [9]. In the present study, permeabilized fibers experiments shown that both capsiate treatments did alter neither the basal nor the maximal respiration rate, which suggests that capsiate does not increase fat oxidation capacity in skeletal muscle. However, it must be point that increased fat oxidation in skeletal muscle has already been observed without any concomitant acceleration of mitochondrial respiration in the case that UCP3 expression increases [33, 34], UCP3 playing a major role in skeletal muscle energy expenditure through the uncoupling of mitochondrial oxygen consumption by the respiratory chain from ATP synthesis [14]. Yet, a single dose of capsiate at 10 mg/kg body weight has been shown to increase UCP3 gene expression by 100% within two hours in mice [9]. Nevertheless, we found that the 2-week capsiate treatments at low or high dose did not affect UCP3 expression thereby ruling out the hypothesis of an increased uncoupling leading to an acceleration of fat oxidation without changes in mitochondrial respiration. Overall, these findings exclude skeletal muscle as a potential candidate mediating the anti-obesity effect of capsiate.

Capsiate treatments caused however marked alterations in muscle bioenergetics in vivo. At rest, both treatments increased intracellular pH (i.e., decreased H^+^ concentration), whereas ATP and ADP contents remained unaffected, and the higher dose of capsiate also reduced basal PCr content. During the whole 6-min fatiguing electrostimulation protocol, these initial differences between control and capsiate group regarding basal pH and PCr content were kept approximately constant, although capsiate treatments did not alter the time-changes in ATP and ADP levels. Intramuscular PCr level is controlled by the CK reaction, which transfers high-energy phosphate from PCr to ADP to form ATP (PCr + ADP + H^+^ ↔ ATP + creatine) [35] and CK/PCr system is considered to play a crucial role in muscle bioenergetics during exercise [36, 37]. A large part of CK activity is ensured by cytosolic CK, which is mainly localized at the fibrillar M-line in association with sarcoplasmic reticulum Ca^2+^ pumps [37]. The remaining activity is due to mitochondrial CK situated in the intermembrane space of mitochondria. The PCr-CK system functions at once as an energy buffer and as an energy carrier in order to maintain ATP homeostasis in exercising muscle [38, 39]. The first function, which is mainly effective at the transition from rest to exercise, allows maintaining ATP pool unchanged despite energy demand might be increased by more than 100-fold. The second function is directly involved in the transport of high-energy phosphate between the site of production (mitochondria) and utilization (myofilaments cross-bridges and ion pumps) of ATP. In the present study, it is therefore surprising that both PCr and H^+^ contents were reduced whereas ADP and ATP levels were not affected because considering the CK reaction equilibrium, any reduction in both PCr and H^+^ contents should result in an increased ADP level in order to maintain ATP homeostasis. At the first glance, these findings suggest that capsiate disturbs the PCr-CK system function. However, it is unlikely that the apparent energy buffer function of PCr-CK system was affected since we found that PCr cost of contraction and ATP level at the onset of the electrostimulation period were not altered by capsiate treatment, thereby indicating that the rate of PCr degradation entirely fits ATP demand for contraction. Furthermore, we found that both capsiate treatments did not affect the rate of PCr resynthesis, an in vivo index of the end-exercise rate of oxidative ATP synthesis [23, 40], hence suggesting that the energy transport function of PCr-CK system was not altered. Interestingly, we found in permeabilized fibers that both capsiate treatments decreased the *K*_m_ of mitochondrial respiration for ADP, which indicates an improvement of mitochondrial sensitivity for ADP [41, 42]. Given that ADP stimulates mitochondrial ATP synthesis through a feedback loop [23, 25], the higher mitochondrial sensitivity for ADP combined with unchanged ADP level should lead to an acceleration of the mitochondrial ATP synthesis. The absence of such an issue herein suggests that capsiate alters the feedback loop of ADP on oxidative metabolism.

Another important finding is that both capsiate treatments led to a reduction in oxidative cost of contraction in the latter phase of the fatiguing protocol, hence indicating that less oxidative ATP was needed for a given amount of force. Capsiate is known to increase Ca^2+^ release from the sarcoplasmic reticulum via the activation of TRPV1 receptors in skeletal muscle [11, 43]. Thus, the reduced cost of contraction could be linked to an enhanced response of contractile elements to Ca^2+^ in combination with an improvement of the kinetics of attachment and detachment of cross-bridges with repeated muscle contractions, as already proposed in exercising muscle of mice receiving a single dose of capsiate [44]. Moreover, it must be underlined that both capsiate treatments did affect neither glycolytic cost of contraction nor the balance between oxidative and anaerobic ATP production, indicating that the improvement of oxidative function did not occur at the expense of other metabolic pathways.

We found also that gastrocnemius muscle volume was larger in groups treated with capsiate at low (+11%) and high (+16%) doses when compared to control animals. This result corroborates those from a previous study showing that 2-week daily administration of capsiate at 10 mg/kg increases gastrocnemius muscle weight by 16% [9], and supports the hypothesis that capsiate promotes muscle growth. One can assume that this hypertrophic effect is mediated by the capsiate-induced activation of TRPV1 receptors, resulting in an increased cytosolic Ca^2+^ level that subsequently triggers the mammalian target of rapamycin (mTOR) [45]. Upregulation of the mTOR signaling pathway actually causes muscle hypertrophy via an increased protein synthesis [46, 47]. Our assumption is supported by previous experiments showing that chronic administration of capsiate at 10 mg/kg during 4 weeks increases protein intake in humans [12]. In addition, it is tempting to speculate that capsiate-induced muscle hypertrophy contributes to the anti-obesity effect of this compound, given that hypertrophied muscle favors fat utilization for growth and maintenance [48]. On the other hand, it is noteworthy that capsiate-induced muscle hypertrophy did affect neither the force-generating capacity nor muscle fatigability, which is consistent with previous works reporting that acute and chronic administration of capsiate do not affect mechanical performance [4, 49].

Overall, the present work demonstrates that, in addition to its anti-obesity effect, chronic administration of capsiate promotes muscle mass gain and improves oxidative metabolism in exercising muscle. These data strengthen capsiate as a natural compound for improving health.
